# Sexual and Reproductive Health among Unmarried Rural-Urban Female Migrants in Shanghai China: A Comparative Analysis

**DOI:** 10.3390/ijerph10083578

**Published:** 2013-08-09

**Authors:** Ying Wang, Wen Yao, Meili Shang, Yong Cai, Rong Shi, Jin Ma, Jin Wang, Huijiang Song

**Affiliations:** 1 School of Public Health Affiliated with School of Medicine, Shanghai Jiaotong University, Shanghai 200025, China; E-Mails: yingwangxun@163.com (Y.W.); shirong61@yahoo.com.cn (R.S.); majin@shsmu.edu.cn (J.M.); 2 Shanghai Hongkou Center for Disease Control and Prevention, Shanghai 200082, China; E-Mail: yw612423@163.com; 3 Shanghai Pudong Sanlin Community Sanitary Service Center, Shanghai 200126, China; E-Mail: lilyshang1984@163.com; 4 Shanghai Pudong Population and Family Planning Management Center, Shanghai 200136, China; E-Mail: wangjinyao2001@yahoo.com.cn

**Keywords:** sexual and reproductive health, female migrant, premarital sex, safe sex

## Abstract

We compared sexual and reproductive health (SRH)-related knowledge, attitude and behavior among unmarried rural-urban female migrants in Shanghai coming from different regions of China. A total of 944 unmarried rural-urban female migrants were recruited from three districts of Shanghai. We used an interviewer-administered structured questionnaire to collect information from each participant and a multivariate logistic regression to examine the association between premarital sex and risk factors. We found the rates of premarital sex, pregnancy and abortion among unmarried rural-urban female migrants were 28.2%, 5.2% and 5.0%, respectively. Participants from the east of China were more likely to engage in premarital sex than those from the mid-west (*p* < 0.001). The analysis showed premarital sex was associated with age, hometown, education, current residential type, knowledge of sexual physiology and safe sex, attitude to SRH and safe sex, and permissive attitude to sex. Unmarried rural-urban female migrants lack SRH related knowledge and the data suggests high levels of occurrence of premarital sex. The results indicate that programs to promote safe sex, especially to those migrants coming from eastern China, should be a priority.

## 1. Introduction

China is one of the fastest growing economies in the World, and migration has contributed to its rapid economic development [1]. In 2010, 260 million, 19.5 percent of China’s total population, were migrants [2]. Most migrants in China are villagers moving from rural areas to urban areas for more job opportunities. According to the estimation from the China National Bureau of Statistics, over 70% of migrants were rural villagers, with the remainder coming from townships and small cities. The Shanghai Bureau of Statistics has no special data about this phenomenon [3]. With the continuous growth of China’s economy, there is no doubt that the migrant population will increase rapidly. Rural-to-urban migrant population in China has been identified as one of the “tipping points” for the epidemic of acquired immunodeficiency syndrome (AIDS) [3,4,5,6,7,8]. This is a universal phenomenon [9,10,11]. One research found that in the European Union between 1999–2006, 35% of 75,021 AIDS cases were migrants. In 2006, 76% of 9,066 HIV infectious females were migrants [12]. Migrants are one of the most vulnerable groups for poor sexual and reproductive health (SRH) [13,14].

In recent years, female migration has risen [4,15,16], and females are considered to be more vulnerable than male migrants [4]. Research indicates that most female migrants migrate to metropolitan cities before marriage, when they are between 15–34 years old [5,12,17,18,19]. In China the Hukou system, which isolates rural from urban citizens, makes it difficult for rural-urban female migrants to access SRH services in the cities [5,7,17,18]. According to family planning agencies in China, the SRH service mainly targets married females and unmarried females are excluded. Several authors report that unmarried female migrants are usually poorly educated, lack SRH-related information, and know little about self-protection. However, female migrants often hold open attitudes to premarital sex [3,15,19,20,21,22]. Unsafe sex leads to unintended pregnancy, abortions, pregnancy-related syndromes, and infectious diseases [17,23]. One study of 1,600 females in China undergoing abortion found 72.9% were migrants and 77.4% were unmarried [24]. A relative survey in Beijing and Shanghai found pregnancy in unmarried migrant women accounted for almost half of total abortions [25,26].

Hence unmarried rural-urban female migrants are a vulnerable group and SRH has become a major concern confronting them. Although several studies have paid close attention to this group [13,20,27], most studies do not consider regional diversity. China is vast and the economic and social differences between the mid-west and eastern regions are great. Unmarried female migrants from different parts of China may have different levels of SRH-related knowledge, attitude, and actions [28]. This study investigates the SRH knowledge, attitude, and behavior of unmarried rural-urban female migrants to provide information for intervention methods.

## 2. Methods

### 2.1. Study Setting

Shanghai is one of the largest metropolitan cities in China. Shanghai’s economic boom has attracted many migrants from throughout China. According to the National Sixth Census (2010), 39% of the Shanghai residents were migrants, 45.8% were female, and more than 30% of these females were unmarried [29].

### 2.2. Sampling Procedure

Three districts of Shanghai were randomly selected firstly. Migrants usually take a physical examination and obtain a medical certificate from medicine centers before employment. We enrolled unmarried female migrants from Medical Centers in each district from July to September 2012. Individuals were eligible if they were unmarried female migrants aged over 16 years because it would be illegal for employers in China to hire any adolescents under 16 years old.

We reviewed previous literature, and found that the reported prevalence of premarital sex among unmarried female migrants varied from 7.8% to 51.8%, average 29% [30]. Assuming a prevalence of the premarital sexual behaviors of 29.0%, an **α** of 0.05, and a relative error of sampling of 0.15*P*, we calculated a sample size of 418. As we collected data from cluster samples, we doubled the sample size to 836 to compensate for any sampling error. Given a response rate of 90%, the required sample size was 920. A total of 1,000 unmarried rural-urban female migrants agreed to participate in this survey and 944 participants aged from 16 to 27 years old completed the questionnaire.

### 2.3. Measures

We used a questionnaire based on the Reproductive Health Questionnaire for Adolescents [31] from the Royal Women’s Hospital in Australia and tested the questionnaire’s reliability and validity in advance [32]. This questionnaire consists of seven questions on socio-demographic characteristics (age, origin of hometown, resident status, income, education, and working place); twenty two questions on the participant’s knowledge about SRH; and fourteen questions about the attitude of the participant to SRH. We also questioned participants on their sexual activity and inquired about other relevant SRH information. Data were collected by face-to-face interviews. Background information about the survey was given to all the participants, and participants were free to ask questions. Each interview lasted approximately 20 min, and participants were paid 20 CNY (USD 3.17) after the interview.

#### 2.3.1. SRH Related Information

The participants were asked 22 questions to assess their knowledge of SRH. We allocated a score of 1 for each “yes” answer and 0 for a “no” or “don’t know” answer. Eight questions (e.g., Does abortion harm the human body?) assessed participant knowledge of sexual physiology and safe sex. A factor analysis found that all eight items had equal coefficients, which indicated the sum of these items could be combined on a composite scale to represent knowledge about sexual physiology and safe sex (Cronbach’s alpha coefficient: 0.79; range: 0–8) where a high score indicated more knowledge of sexual physiology and safe sex. Similarly, fourteen questions (e.g., Can AIDS be spread by kissing someone with AIDS?) assessed STI (sexually transmitted disease)/AIDS knowledge. A factor analysis found that the sum of these 14 items could be combined on composite scale representing knowledge about STI/AIDS (Cronbach’s alpha coefficient: 0.74; range: 0–14), where a high score indicated greater knowledge of STI/AIDS.

#### 2.3.2. SRH Related Attitude

SRH related attitude consisted of 13 questions. All the questions were constructed from answers on a 5-point Likert scale (1 completely agree to 5 completely disagree) [33]. Four items (e.g., What do you think of married women who have sexual intercourse with males besides their husbands?) measured permissive attitudes to sex. A factor analysis found that all four items had equal coefficients, which indicated the sum of these items could be combined on a composite scale to represent permissive attitudes to sex. We added the scores for each item to get a total score (Cronbach’s alpha coefficient: 0.82; range: 4–20), where lower scores indicated a more permissive attitude to sex.

Five questions (e.g., What do you think of one-night stands?) assessed attitude to SRH and safe sex. Similarly, we added the score for each item to assess participant’s attitude to SRH and safe sex (Cronbach’s alpha coefficient: 0.71; range: 5–25). 

The rest four questions measured self-efficacy (self-efficacy is the measure of one's own ability to complete tasks and reach goal.) to safe sex (e.g., Do you think you should use a condom during sexual intercourse?). In the same way, we added the score for each item to represent participant’s self-efficacy to safe sex (Cronbach’s alpha coefficient: 0.72; range: 4–20).

#### 2.3.3. SRH Related Behaviors

SRH-related behaviors were assessed by five questions (Did you have premarital sexual intercourse? If you did, did you have a premarital pregnancy? Have you had a premarital abortion? Have you had reproductive tract infection? Do you use condoms consistently?).

### 2.4. Data Analysis

Data were double entered with Epidata 3.0. We used the Statistical Program for Social Sciences (SPSS, version 20.0) software to analyze data. Descriptive statistics, such as means, standard deviations, frequencies, and percentages were used to assess the characteristics, SRH related information, attitude, and behaviors of unmarried rural-urban female migrants. The differences in characteristics, SRH related information, attitude, and behaviors between migrants from the mid-west and the east were tested with χ^2^ tests or Student’s *t* test. Independent variables were tested for normality prior to multivariate analysis. A dependent variable (having premarital sex) was categorized into “yes” or “no” answers and a binary logistic regression was used to identify determinants of premarital sex. We entered the variables into the regression model using a forward stepwise approach to prevent co-linearity and set the significance levels at 0.05.

## 3. Results

### 3.1. Characteristics of Participants

A total 944 participants completed the questionnaire (Table 1). They were, on average, 21.3 years old (SD = 2.3; range: 16 to 27); 702 (74.4%) were from the mid-west, and 242 (25.6%) were from the east; most were more than 20 years old (the legitimate marriage age for females). 60.7% had finished senior high school; 77.6% reported monthly income less than CNY 3,000 (USD 481.7), 68.2% worked in the service industry and 73.7% lived in dormitory accommodation. The distribution of age, education and other characteristics of unmarried female migrants from both areas was similar (*p* > 0.05).

**Table 1:** Socio-demographic characteristics of unmarried rural-urban female migrants from mid-west and east China.

Characteristics		Mid-west (*n* =702) n (%)	East (*n* =242) n (%)	Total (*n* = 944) n (%)	χ^2^	*p*
Age(years)						
	16–20	218 (31.1%)	86 (35.5%)	304 (32.2%)	1.657	0.198
	≥21 *	484 (68.9%)	156 (64.5%)	640 (67.8%)		
Education						
	Middle school	271 (38.6%)	100 (41.3%)	371 (39.3%)	0.557	0.455
	Senior high school	431 (61.4%)	142 (58.7%)	573 (60.7%)		
Monthly income (CNY **)						
	<3,000	555 (79.1%)	178 (73.6%)	733 (77.6%)	3.144	0.076
	≥3,000	147 (20.9%)	64 (26.4%)	211 (22.4%)		
Occupation						
	Service	482 (68.7%)	162 66.9%)	644 (68.2%)	0.245	0.620
	Manufacturing	220 (31.3%)	80 (33.1%)	300 (31.8%)		
Current residential type						
	Rental	175 (24.9%)	73 (30.2%)	248 (26.3%)	2.548	0.110
	Dormitory	527 (75.1%)	169 (69.8%)	696 (73.7%)		

* In China, 20 years is youngest legal age for females to marry; ** CNY: Chinese Yuan; 6.228 CNY = 1 USD (15 November 2012).

### 3.2. Comparison of SRH Related Information, Attitude and Behaviors

Most unmarried rural-urban female migrants had little knowledge about SRH. The mean score for sexual physiology and safe sex information was 4.9, and STI and HIV/AIDS knowledge was 7.4 (Table 2). Migrants from the east scored higher (5.7 and 7.9) than those from the mid-west (4.6 and 7.3) on SRH related information, which meant that they knew more about SRH. On SRH related attitude questions, migrants from the east scored higher (15.2) than migrants from the mid-west (14.7) indicating they held more permissive attitude to sex, and migrants from the east scored higher when talking about attitudes to SRH, safe sex, and self-efficacy to safe sex. Five items were designed to evaluate SRH related behaviors, and 28.2% of the participants reported premarital sexual intercourse. We found migrants from the east were more likely to experience premarital sex than those from the mid-west; 5.2% of these unmarried female migrants reported becoming pregnant, 5.0% had premarital abortion, and 8.1% had experienced reproductive tract infection. These data indicate that the rates of premarital sex, unintended pregnancy and abortion were higher for migrants from the east than the mid-west.

**Table 2:** Comparison of SRH related information, attitude, and behaviors among unmarried rural-urban female migrants from mid-west and east China.

Items		Midwest	East	Total	*t/*χ^2^	*p*
		(*n* = 702)	(*n* = 242)	(*n* = 944)		
SRH related information (Mean, SD)						
	Sexual physiology and safe sex related knowledge	4.64 ± 1.94	5.66 ± 1.83	4.90 ± 1.97	*7.183*	*<0.001*
	STI /AIDS related knowledge	7.26 ± 2.85	7.86 ± 2.91	7.41 ± 2.87	*2.804*	*0.005*
SRH related attitude (Mean, SD)						
	Permissive attitude to sex	15.19 ± 2.80	14.70 ± 2.97	15.07 ± 2.85	*2.333*	*0.020*
	Attitude to SRH and safe sex	22.46 ± 2.41	22.81 ± 2.66	22.55 ± 2.48	*1.894*	*0.058*
	Self-efficacy to safe sex	14.63 ± 2.62	15.35 ± 2.27	14.81 ± 2.56	*4.079*	*<0.001*
SRH related behaviors						
	Experience of premarital sexual intercourse (n, %)	152 ± 21.7	114 ± 47.1	266 ± 28.2	*57.618*	*<0.001*
	Experience of premarital pregnancy (n, %)	26 ± 3.7	23 ± 9.5	49 ± 5.2	*12.303*	*<0.001*
	Experience of premarital abortion (n, %)	25 ± 3.6	22 ± 9.1	47 ± 5.0	*11.631*	*<0.001*
	Experience of Reproductive tract infection (n, %)	43 ± 6.1	33 ± 13.6	76 ± 8.1	*13.715*	*<0.001*
Consistent condom use among participants who had reported premarital sex (n, %)		82 ± 53.9	57 ± 50.0	139 ± 52.3	*0.407*	*0.524*

### 3.3. Predictors Associated with Premarital Sexual Intercourse

A multivariate logistic regression model indicated seven factors were associated with premarital sex (age, hometown, education, current residential type, knowledge of sexual physiology and safe sex, attitude to SRH and safe sex, and permissive attitude to sex). Unmarried female migrants older than 20 were more likely to have premarital sexual intercourse than migrants less than 20 years (Table 3), and migrants from the east were more likely to have premarital sexual intercourse than those from the mid-west. Better-educated migrants were less likely to have premarital sexual intercourse and migrants living in rental instead of dormitory accommodation were more likely to have premarital sexual intercourse. Knowledge of sexual physiology and safe sex, attitude to SRH and safe sex and permissive attitude to sex were also important determinants of premarital sexual intercourse.

**Table 3:** Logistic regression models predicting premarital sexual intercourse among unmarried rural-urban female migrants from mid-west and east China (*N* = 944).

Variable	Total (*n* = 944)		Mid-west (*n* = 702)		East (*n* = 242)	
	OR (95% CI)		OR (95% CI)		OR (95% CI)	
Age group (>20 *vs*. <20 years)	3.66 **	(2.33–5.75)	3.53 **	(2.07–6.22)	5.27 **	(2.25–12.32)
Hometown (east *vs*. mid-west)	2.82 **	(1.92–4.14)	-	-	-	-
Education (≤middle school *vs*. high school)	2.07 **	(1.40–3.05)	1.66 *	(1.06–2.62)	4.31 **	(1.88–3.05)
Current residential type (rental *vs*. dormitory)	2.03 **	(1.39–2.95)	2.33 **	(1.50–3.61)	-	-
Knowledge of sexual physiology and safe sex	1.47 **	(1.32–1.64)	1.50 **	(1.31–1.70)	1.51 **	(1.22–1.88)
Attitude to SRH and safe sex	1.23 **	(1.14–1.34)	1.19 *	(1.08–1.31)	1.37 **	(1.16–1.62)
Permissive attitude to sex	0.72 **	(0.68–0.77)	0.79 **	(0.73–0.86)	0.56 **	(0.47–0.65)

SRH = sexual and reproductive health; CI = confidence interval; OR = odds ratio; *p* value: * <0.05 ** <0.001.

## 4. Discussion

Here we found that 28.2% of rural-urban female migrants had premarital sexual intercourse, which is a higher rate than that reported in previous studies [27]. Although this figure may be lower than that in some developed countries [6,10], the rate is indicative of the profound social, economic and cultural change experienced in China over the last three decades [18]. Dramatic social changes have reshaped people’s traditional ideas, and unmarried rural-urban female migrants are now more likely to hold favorable attitudes to premarital sexual intercourse, and our data, in this respect, support findings by other researchers [3]. However, migrants do not have enough knowledge and affordable access to SRH service, which leads to serious problems, such as unexpected pregnancy, abortion, and STIs. 

In our study, the rates of unwanted pregnancy and unintended abortion among unmarried female migrants were 5.2% and 5.0%, which indicates that most migrants who become pregnant chose to end pregnancy by abortion. Abortion may be chosen because they may have no money or time to bring up a baby, or their relationship with their partner might not be stable. This finding is consistent with a previous study of unmarried graduate students [34].

This finding indicates intervention to promote safe sex is urgently needed for female migrants. Unsafe sex practice (for example, inconsistent condom use) may have serious consequences besides unwanted pregnancy and abortion.

Although our findings were a little lower than other reports [30], we still found that the rate of premarital sexual intercourse in our sample population was much higher than the rate reported for adolescents between 16–26 years in China [17,27,34,35,36]. This was probably because most of these unmarried female migrants dropped out of school at a very young age while other adolescents would attend senior high school or college. Most of the adolescents in school still have a strong social attachment to their classmates, friends, and parents. They live up to higher expectations, so they have to work very hard and couldn’t afford to get pregnant. Otherwise, they couldn't continue to pursue their studies. On the contrary, these unmarried female migrants are separated from their home and peers [5,8]. Unlike college students who have already got a ticket to the future, they don’t have too much expectations of themselves. Finding a fine young man to get married might be a better way out for these unmarried female migrants, so they tend to rely on men not only emotionally, but also financially, which might reduce moral constraints to have premarital sex [3,5,16,17]. At the same time, they left school so early and couldn’t get enough knowledge of SRH and self-protection. Evidence also showed that it was more difficult for adolescents who have dropped out of school to get access to SRH-related information [37]. In other words, it was more likely for these unmarried female migrants to have risky sexual behavior than average adolescents. Risky sexual behavior leads to unwanted pregnancy, unintended abortion, reproductive tract infection and STDs including HIV [13,18,38]. They are more fragile, and this is the exact reason why we choose unmarried female migrants as our target population.

In this study, we compared unmarried rural-urban female migrants from the mid-west and the east. Although migrants from the east made more money than those from the mid-west, both groups presented similar socio-demographic characteristics, such as age, education, occupation, and current residential type, but migrants from the east scored higher for SRH-related information, and we infer that eastern migrants may have more access to sex related information because the eastern economy is more developed than that in the mid-west. Mid-western migrants come from a region that is economically backward and also has limited channels of information, including SRH information. Eastern migrants were more permissive and scored higher for self-efficacy to safe sex. This implies eastern migrants were more likely to protect themselves when confronted with sex related issues. Eastern migrants were also more likely to have premarital sexual intercourse, hence, rates of premarital pregnancy, abortion, and reproductive tract infection were all higher. 

We also discovered that factors such as age, hometown, and education were important predictors of premarital sex for unmarried female migrants. These predictors are similar to the findings of previous research focusing on social and demographic characteristics affecting SRH service use [17]. Further, it was interesting to find that the accommodation type (rental or dormitory) changed premarital sex behavior for mid-western but not eastern migrants. The difference may arise because Shanghai is in eastern China. Mid-western migrants were far from home, the links between the migrants and their families were weak, and life would be easier (not only economically but also emotionally) with a male partner. In contrast, eastern women were near to home, and were less likely to rent a house than mid-western migrants. This may explain why accommodation type was a predictor of premarital sex behaviors for mid-western but not for eastern migrants.

Our study has several limitations. First, this was a cross-sectional survey and this method limits the exploration of influential factors and thus the inference of causal inferences. Second, all participants were chosen from women attending a Medical Center and the sample excluded unemployed, unmarried female migrants. Although most of these unmarried female migrants would find a job either by themselves or through their fellow-villagers in Shanghai, the sample population should therefore be expanded and cover the objects who could not find a job in the future studies. Last, survey data were self-reported and participants may not have been completely truthful because premarital sex behavior is a sensitive topic in mainland China. Bias of self-reported data was inevitable, however, reliability and validity studies indicated good test-retest and fit of the results for the questionnaire we used [32].

Despite these limitations, our findings show that unmarried rural-urban female migrants knew little about SRH, have a permissive attitude, and it was easy for them to have premarital sexual intercourse. These results indicate a need to increase intervention to promote safe sex. The huge cultural differences between the east and mid-west of China indicate we should focus on different factors when we conduct interventions. More SRH information should be delivered to those from the mid-west since that they know less than those from the east.

## 5. Conclusions

Unmarried female migrants experience high rates of premarital sex while they know little about SRH. Safe sex should be promoted to reduce unwanted pregnancy and abortion. Mid-western unmarried migrants, especially, have poor SRH related knowledge and need more SRH related information.
